# Toward the defined and xeno-free differentiation of functional human pluripotent stem cell–derived retinal pigment epithelial cells

**Published:** 2011-02-22

**Authors:** Hanna Vaajasaari, Tanja Ilmarinen, Kati Juuti-Uusitalo, Kristiina Rajala, Niina Onnela, Susanna Narkilahti, Riitta Suuronen, Jari Hyttinen, Hannu Uusitalo, Heli Skottman

**Affiliations:** 1 Regea - Institute for Regenerative Medicine, University of Tampere, Tampere, Finland; 2 Department of Biomedical Engineering, Tampere University of Technology, Tampere, Finland; 3 Department of Eye, Ear, and Oral Diseases, Tampere University Hospital, Tampere, Finland; 4 SILK, Department of Ophthalmology, University of Tampere, Tampere, Finland

## Abstract

**Purpose:**

The production of functional retinal pigment epithelium (RPE) cells from human embryonic (hESCs) and human induced pluripotent stem cells (hiPSCs) in defined and xeno-free conditions is highly desirable, especially for their use in cell therapy for retinal diseases. In addition, differentiated RPE cells provide an individualized disease model and drug discovery tool. In this study, we report the differentiation of functional RPE-like cells from several hESC lines and one hiPSC line in culture conditions, enabling easy translation to clinical quality cell production under Good Manufacturing Practice regulations.

**Methods:**

Pluripotent stem cells were cultured on human fibroblast feeder cells in serum-free medium. The differentiation toward RPE was induced by removing basic fibroblast growth factor and feeder cells from the serum-free conditions. RPE differentiation was also achieved using xeno-free and defined culture conditions. The RPE cell morphology and pigmentation of the cells were analyzed and the expression of genes and proteins characteristic for RPE cells was evaluated. In vitro functionality of the cells was analyzed using ELISA measurements for pigment epithelium derived factor (PEDF) secretion and phagocytosis of photoreceptor outer segments (POS). The integrity of the generated RPE layers was analyzed using transepithelial electric resistance measurements.

**Results:**

We generated putative RPE cells with typical pigmented cobblestone-like morphology. The expression of RPE-specific markers was confirmed at the gene and protein level. The differentiated cells were able to phagocytose POS and secrete PEDF characteristic of native RPE cells. In addition, cultured cells formed a polarized epithelium with high integrity and exhibited excellent transepithelial electric resistance values, indicating well established, tight junctions. Moreover, we introduced an improved method to generate functional putative RPE cells without xeno-components under defined conditions.

**Conclusions:**

We have developed a progressive differentiation protocol for the production of functional RPE-like cells from hESCs and hiPSCs. Our results demonstrate that putative hESC-RPE and hiPSC-RPE express genes and proteins characteristic for RPE cells, as well as being able to phagocytose POS and secrete PEDF. Furthermore, our results show that RPE-like cells can be differentiated in xeno-free and defined culture conditions, which is mandatory for Good Manufacturing Practice-production of these cells for clinical use.

## Introduction

Retinal pigment epithelium (RPE) is an epithelial cell monolayer located between the neural retina and choriocapillaris. RPE provides essential support for the long-term preservation of retinal integrity and visual functions by absorbing stray light, regenerating visual pigment, supplying nutrients, secreting growth factors, and phagocytosing the shed photoreceptor outer segments (POS) [1]. Dysfunctional RPE causes impairment and death of the photoreceptor cells, leading to deterioration or total loss of vision. These mechanisms play an important role in the pathogenesis of retinal diseases like age-related macular degeneration (AMD), which is the leading cause of blindness in the developed world [2]. Intravitreal vascular endothelial growth factor antagonism has been shown to prevent vision loss and even improve visual acuity in patients with neovascular AMD in the early course of the disease. However, in advanced cases of exudative AMD, as well as in the most common form of AMD, nonexudative AMD, there is no satisfactory cure. Even though vascular endothelial growth factor antagonists are effective, intravitreal injections are needed and this causes high costs for the health care system while exposing the patients to complications such as endophthalmitis, myocardial infarction, or stroke [3]. In the search for more a comprehensive therapy for AMD, tissue engineering and cell transplantation are among the most promising candidates. Several cell sources have been considered [4-9].

**Figure 1:**
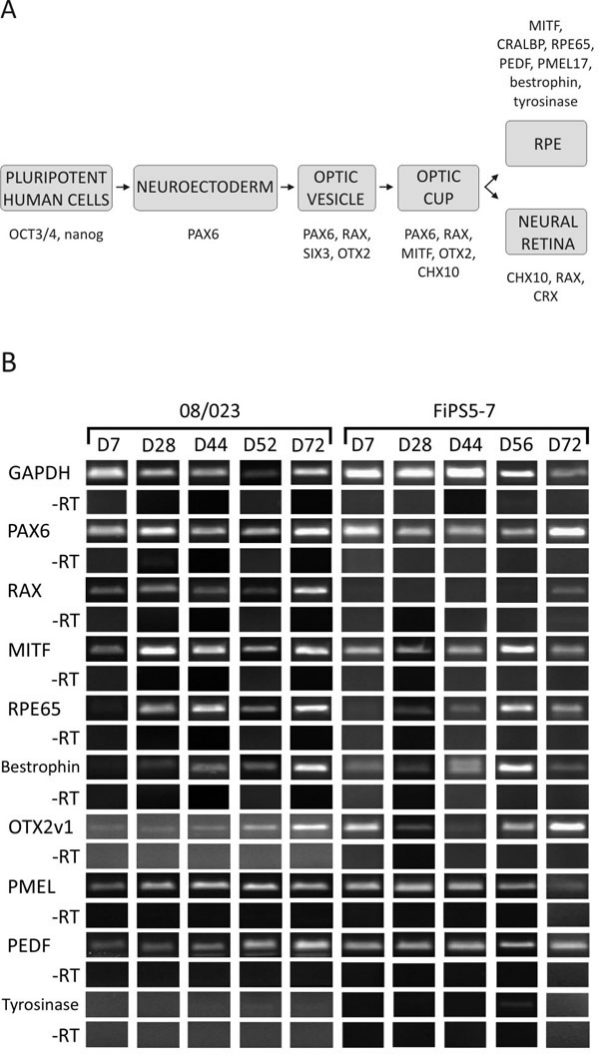
Differentiation of human pluripotent stem cells toward retinal pigment epithelium cells. **A**: A schematic representation of retinal pigment epithelium (RPE) cell differentiation during retinal development. **B**: Reverse transcription (RT)–PCR analysis of typical genes for retinal development expressed during putative RPE differentiation of the human embryonic stem cell (hESC) line Regea 08/023 and human induced pluripotent stem cell (hiPSC) line FiPS 5–7 at sequential time points on D7–D72.

The cellular origin of the retina is exclusively ectodermal. During development, the first morphological correlates of the eye are the optic pit and optic vesicle with the retinal progenitor cells, and eventually the optic cup with two distinct layers: the RPE originating from the outer layer, and the neural retina from the inner layer (Figure 1A). The organization of the vertebrate retina into well defined layers is a result of a complex series of developmental processes influenced by a variety of intrinsic and extrinsic factors. Retinal progenitor cells give rise to all retinal cell types such as RPE cells, photoreceptor cells (rods and cones), bipolar cells, ganglion cells, amacrine and horizontal cells, astrocytes, and Müller glial cells [10,11].

Human pluripotent stem cells may serve as an unlimited source of RPE cells for transplantation. Several groups have reported successful RPE differentiation originating from human embryonic stem cells (hESCs) and human induced pluripotent stem cells (hiPSCs; summarized in Table 1). It is widely acknowledged that properties of the existing pluripotent cell lines vary depending on cell line and culture conditions [12-14]. Hence, it is necessary to test RPE differentiation methods and their capacities using several different cell lines. The hESC-RPE and hiPSC-RPE have been shown to delay photoreceptor loss and improve visual functions after subretinal transplantations in animal models. However, transplanted cells disappear within first months after transplantation [4,15-17]. Cell survival needs to be improved to maintain the total functionality of the retina. In addition, these cells have potential in disease modeling and drug discovery, where they are superior to widely used cell lines such as human retinal pigment epithelial cell line (ARPE-19), at least resembling more closely their native counterparts according to their gene expression [1].

**Table 1:** Summary of published retinal pigment epithelium (RPE) differentiation methods.

**Reference**	**Number of cell lines**	**Feeder cells / matrix for undifferentiated cells**	**Serum / serum replacement for** **a) undifferentiated cells** **b) differentiation**	**Differentiation matrix**	**Serum free^1^**	**Xeno-free^2^**
[1]	hESC (11)	MEF	a) KO-SR, plasmanate	MEF, Gelatin; Feeder-free; EB	-	-
** **	** **	** **	b) KO-SR, (plasmanate, FBS)	** **	** **	** **
[17]	hESC (18)	MEF	a) KO-SR, plasmanate	MEF, Gelatin	-	-
** **	** **	** **	b) KO-SR, plasmanate, FBS	** **	** **	** **
[22]	hESC (2)	MEF	a) KO-SR	SFEB, Poly-D-Lysine-Laminin-Fibronectin	+	-
** **	** **	** **	b) KO-SR	** **	** **	** **
[50]	hESC (1)	MEF	a) KO-SR, FBS	Mouse PA6 cells, Human Bruch’s membrane, Matrigel™	-	-
** **	** **	** **	b) KO-SR	** **	** **	** **
[37]	hESC (2)	MEF	a) KO-SR	MEF, Matrigel™	+	-
** **	** **	** **	b) KO-SR	** **	** **	** **
[51]	hESC (1)	MEF	a) KO-SR	MEF, Matrigel™	+	-
** **	** **	** **	b) KO-SR	** **	** **	** **
[52]	hiPSC (2)	mouse and human cells	a) FBS, KO-SR	SFEB, Poly-D-Lysine-Laminin-Fibronectin	-	-
** **	** **	** **	b) KO-SR	** **	** **	** **
[21]	hESC (1) hiPSC (4)	MEF, SNL	a) KO-SR	SFEB, Poly-D-Lysine-Laminin-Fibronectin	+	-
** **	** **	** **	b) KO-SR	** **	** **	** **
[32]	hESC (1) hiPSC (4)	hFF, MEF, MatrigelTM	a) KO-SR	Gelatin	-	-
** **	** **	** **	b) KO-SR, FBS	** **	** **	** **
[16]	hESC (4)	hFF	a) KO-SR	Floating clusters, Laminin	+	-
** **	** **	** **	b) KO-SR	** **	** **	** **
[34]	hESC (1) hiPSC (4)	MEF	a) KO-SR	Free-floating aggregates, Laminin	+	-
** **	** **	** **	b) KO-SR, NIM, RDM	** **	** **	** **
[33]	hiPSC(1)	MEF	a) KO-SR	MEF, Gelatin	-	-
** **	** **	** **	b) FBS	** **	** **	** **
[53]	hESC (2)	MatrigelTM	a) MEF-CM	Collagen-Laminin	-	-
** **	** **	** **	b) Serum-free + FBS	** **	** **	** **

^1^No human serum (HS), fetal bovine serum (FBS) or plasmanate used in any stage of pluripotent stem cell cultures or during differentiation. ^2^No animal cells, BSA (BSA)/FBS or other xeno components used in any stage of pluripotent stem cell cultures or during differentiation. Abbreviations: hESC represents human embryonic stem cell; hiPSC represents human induced pluripotent stem cell; MEF represents mouse embryonic fibroblast; KO-SR represents KnockOut Serum Replacement; EB represents embryoid body; SFEB represents serum-free floating culture of embryoid body-like aggregates; SNL represents a mouse fibroblast STO cell line; NIM represents chemically defined neural induction medium; MEF-CM represents mouse embryonic fibroblast conditioned medium.

Current differentiation methods for RPE cells mainly rely on spontaneous differentiation processes favoring neuroectodermal lineage, which is characteristic of hESCs [18-20]. RPE differentiation efficiency has been recently enhanced with a prolonged culture period and cell culture supplements. Studied supplements include: nicotinamide (NIC), Activin A, transforming growth factor beta (TGFβ) [16], Wnt signaling inhibitor casein kinase I inhibitor (CKI)-7, dickkopf-related protein-1 (Dkk-1), Lefty-A, fibroblast growth factor antagonist Y-27632, and nodal signaling inhibitor SB431542 [21,22]. Regardless of the improvement of the differentiation efficacy, reaching a sufficient amount of maturated cells with RPE characteristics still demands long-term differentiation processes.

Xeno-products and undefined factors used in the differentiation processes pose further challenges, because animal-derived components may carry factors such as sialic acid or Neu5Gc, causing unwanted immunogenicity of the cells [23,24] or even animal pathogens. Fetal bovine serum (FBS) is widely used, at least in some stages of the culture of RPE cells (Table 1). KnockOut™ Serum Replacement (KO-SR), used to replace FBS in many laboratories, still contains BSA (BSA) and bovine transferrin [25]. In addition, most of the published differentiation methods utilize the culture environment produced by mouse embryonic fibroblast (MEF) cells widely used as feeder cells for hESCs and hiPSCs. It has been suggested that MEFs may even favor the spontaneous differentiation process of RPE cells [26]. Recently, Idelson and coworkers published RPE differentiation from hESCs cultured on human foreskin fibroblasts (hFFs) and by using KO-SR in differentiation medium [16], providing the first important steps toward a defined and xeno-free culture and differentiation process.

In this study, we demonstrate the differentiation potential toward RPE cells of four hESC lines and one hiPSC line, and further give the molecular and functional characterization of these cells. Human ESC- and hiPSC-derived pigmented cells show a typical RPE morphology and express genes and proteins that are characteristic for RPE. The monolayer of putative RPE cells demonstrates functional integrity analyzed by transepithelial electric resistance (TEER). Additionally, the cells phagocytose POS and secrete pigment epithelium-derived factor (PEDF), which is crucial for functional RPE. In the current study, we introduce a defined and xeno-free RPE cell differentiation method based on our previously developed culture system for undifferentiated hESCs and hiPSCs [27]. Such a culture system will be needed in the production of RPE cells following Good Manufacturing Practice for clinical applications.

## Methods

### Cell lines

We evaluated the RPE differentiation capacity of four hESC lines: Regea 08/023 (46, XY), Regea 08/017 (46, XX), Regea 08/056 (46, XX), and Regea 08/013 (46, XY), which were previously derived in our laboratory, and one hiPSC line—FiPS 5–7—derived by Professor Otonkoski’s group at the University of Helsinki, Finland [27,28]. The hiPSC line FiPS 5–7 was generated from human fibroblasts using four factors: OCT3/4 (POU5F1), SOX2, nanog, and LIN28 [27]. Transgene silencing was confirmed with quantitative reverse transcription PCR (qRT–PCR) [29]. The cell lines were cultured at +37 °C in 5% CO_2_ on mitotically inactivated (γ-irradiated, 40 Gy) hFF (36,500 cells/cm^2^; CRL-2429; American Type Culture Collection, ATCC, Manassas, VA) with basic hESC culture medium. Basic hESC culture medium consisted of Knockout Dulbecco’s Modified Eagle Medium containing 20% KO-SR, 2 mM Glutamax, 0.1 mM 2-mercaptoethanol (all from Invitrogen, Carlsbad, CA), 1% Minimum Essential Medium nonessential amino acids, 50 U/ml penicillin/streptomycin (both from Cambrex Bio Science, Walkersville, MD), and 8 ng/ml human basic fibroblast growth factor (bFGF; R&D Systems Inc., Minneapolis, MN). The culture medium was changed six times a week. Undifferentiated colonies were passaged manually once a week on the top of hFFs.

### Retinal pigment epithelium differentiation in serum-free conditions

To induce spontaneous RPE differentiation, KO-SR concentration was reduced from 20% to 15% and bFGF was removed from the basic hESC culture medium (described above). This modified medium was called RPEbasic. The cells were cut manually between days 1 and 7 after initiation of differentiation onto low cell bind six-well plates (Nalge NUNC, Tokyo, Japan), where the cells formed floating aggregates. While the cells were cultured on hFFs, the culture medium was changed six times a week (2 ml/35×10 mm polystyrene dish), but for floating cell aggregates, the culture medium change was reduced to three times a week (3 ml/well in a six-well plate). Floating aggregates were passaged mechanically with a scalpel to make gas and nutrient exchange possible. To gain purer RPE populations, the areas with pigmented cells were isolated manually with a scalpel and subsequently dissociated with 1× Trypsin-EDTA (Lonza, Walkersville, MD). The cells were seeded either on Collagen IV (Sigma-Aldrich, St. Louis, MO)-coated 24 well plates for RT–PCR analyses or on permeable Collagen IV–coated 0.3 cm^2^ BD Biocoat culture plate inserts (BD Biosciences, San Jose, CA). After the seeding, the cells were not subcultured.

### Retinal pigment epithelium differentiation in xeno-free and defined conditions

**Figure 2:**
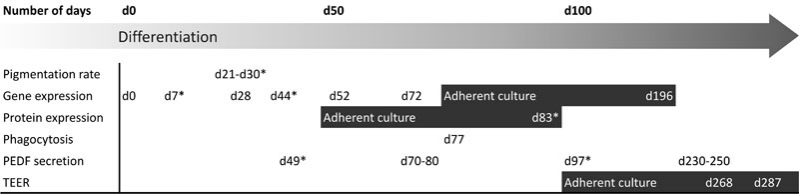
A schematic illustration of implementation of the study. Analyses which were done from the RPEregES condition besides the RPEbasic condition are marked with asterisks (*). Analyses were done from floating aggregate cultures unless marked with gray boxes for adherent culture.

RPE differentiation was studied using a previously developed xeno-free and defined culture medium formulation (RegES) [27] as base. For RPE differentiation, bFGF and retinol were excluded from the published medium formulation (RPEregES). Otherwise, the differentiation method was same as described above (RPEbasic). Analyses conducted in this study from cells differentiated using RPEbasic and RPEregES methods are summarized in Figure 2.

### Analyses of pigmentation rates

The appearance of the first pigmented cells was followed daily. Percentage of pigment containing cell aggregates from the total amount of aggregates was counted between days 21 and 30 after induction of the differentiation. In addition, the day of the first pigmented cell’s appearance was recorded.

### Reverse transcription polymerase chain reaction

RNA samples were collected from cell cultures at five different time points, on days (D; D7, D28, D44, D52, D72) and, in addition, on D196, from selected RPE cells (RPEbasic). From cells differentiated using RPEregES, RNA samples were collected on D7 and D44. Total RNA was extracted using NucleoSpin XS-kit (Macherey-Nagel, GmbH & Co, Düren, Germany) according to the manufacturer’s instructions. Briefly, the cells were lysed in 102 µl of RA1-TCEP mixture. Five µl of Carrier RNA working solution was added to the lysate. The sample was filtrated through the NucleoSpin® Filter Column. One hundred µl of 70% ethanol was added to the lysate and RNA bound to the NucleoSpin® RNA XS Column. The column was desalted using membrane desalting buffer. DNA was digested using DNase reaction mixture. rDNase was inactivated with RA2 buffer and silica membrane washed twice with RA3 buffer. Pure RNA was eluated in 10 µl H_2_O. The RNA concentration and quality were assessed with a NanoDrop 1000 spectrophotometer (NanoDrop Technologies, Wilmington, DE). RNA (40 ng) was reverse-transcribed using MultiScribe Reverse Transcriptase (Applied Biosystems, Foster City, CA) according to the manufacturer’s instructions in the presence of RNase inhibitor. Briefly, 10 µl reaction consisted of: 2.0 µl 10× RT Buffer, 0.8 µl 100 mM dNTP Mix, 2 µl 10 µM gene specific primers, 1.0 µl MultiScribeTM ReverseTranscriptase, 1.0 µl RNase Inhibitor, 11.2 µl Nuclease-free H2O, and 2 µl total RNA (20 ng/µl). The cDNA synthesis was performed as follows: 10 min at 25 °C, 120 min at 37 °C, 5 s at 85 °C. In addition, genomic control reactions excluding the enzyme (-RT) for each RNA sample were performed. CDNA was used as a template in a following PCR reaction, which was performed using 5 U/μl Taq DNA Polymerase (Fermentas, Thermo Fisher Scientific Inc., Leicestershire, UK) with 5 μM primers specific for particular genes (Biomers.net GmbH, Söflinger, Germany; Table 2).

**Table 2:** Reverse-transciptase (RT)–PCR Primer sequences.

** **	**Primer sequences (5′ > 3′)**		** **
**Gene**	**Forward**	**Reverse**	**Tann**
*GAPDH*	GTTCGACAGTCAGCCGCATC	GGAATTTGCCATGGGTGGA	55
*PAX6*	AACGACACAGCCCTCACAAACA	CGGGAACTTGAACTGGAACTGAC	60
*RAX*	CTGAAAGCCAAGGAGCACATC	CTCCTGGGAATGGCCAAGTTT	55
*MITF*	AAGTCCTGAGCTTGCCATGT	GGCAGACCT TGGTTTCCAA	52
*RPE65*	TCCCCAATACAACTGCCACT	CACCACCACACTCAGAACTA	52
bestrophin	GAATTTGCAGGTGTCCCTGT	ATCCTCCTCGTCCTCCTG AT	55
*OTX2v1*	GGGCCCTGGGCTTCTTGTCC	ATTGGCCACTTGTTCCACTC	52
*PMEL*	GTGGTCAGCACCCAGCTTAT	GAGGAGGGGGCTATTCTCAC	52
*PEDF*	AGCTCGCCAGGTCCACAAAG	TGGGCAATCTTGCAGCTGAG	60
tyrosinase	TGCCAACGATCCTATCTTCC	GACACAGCAAGCTCACAAGC	52
*SOX10*	AGCCCAGGTGAAGACAGAGA	AGGAGAAGGCCGAGTAGAGG	55
alphafetoprotein	GCTGGATTGTCTGCAGGATGGGGAA	TCCCCTGAAGAAAATTGGTTAAAAT	55
alpha carciac actin	GGAGTTATGGTGGGTATGGGTC	AGTGGTGACAAAGGAGTAGCCA	55
*nanog*	TGAAATGTCTTCTGCTGAGAT	GTTCAGGATGTTGGAGAGTTC	55
*OCT 3/4*	CGTGAAGCTGGAGAAGGAGAAGCTG	AAGGGCCGCAGCTTACACATGTTC	62

Abbreviations: Glyceraldehyde 3-phosphate dehydrogenase (*GAPDH*), paired box gene 6 (*PAX6*), retina and anterior neural fold homeobox (*RAX*), microphthalmia-associated transcription factor (*MITF*), anti-retinal pigment epithelium-specific 65 kDa protein (*RPE65*), orthodenticle homeobox 2 variant 1 (*OTX2v1*), premelanosome protein (*PMEL*), pigment epithelium-derived factor (*PEDF*), sex determining region Y-box 10 (*SOX10*).

PCR reactions were performed in PCR MasterCycler ep gradient (Eppendorf AG, Hamburg, Germany) as follows: after the hot start denaturation at 95 °C for 3 min and 38 cycles of denaturation at 95 °C for 30 s, 30 s annealing (annealing temperature depended on the used primer pair, see Table 2) and extension at 72 °C for one min, followed by final extension at 72 °C for 5 min. Annealing temperatures and primer sequences are presented in Table 2. PCR products were analyzed on 2.0% agarose gels with a 50 bp DNA ladder (MassRuler^TM^ DNA Ladder Mix; Fermentas). The bands were visualized with Quantity one 4.5.2. Basic program (Bio-Rad Laboratories, Inc., Hercules, CA).

### Quantitative reverse transcription polymerase chain reaction

To further analyze octamer-binding transcription factor (*OCT)3/4* expression in the selected hiPSC-RPE sample after D196 differentiation, we compared expression levels with the sample from undifferentiated cells of the same cell line using qRT–PCR. In addition, we analyzed retina and anterior neural fold homeobox (*RAX)* expression in FiPS 5–7 with qRT–PCR. RNA isolation and cDNA synthesis were done as described above, except that cDNA was synthesized from 200 ng of RNA. FAM-labeled TaqMan Gene Expression Assays (Applied Biosystems) were used to analyze expression of the pluripotency marker, *OCT3/4* (Hs00999632_g1), and *RAX* (Hs00429459_m1). Triplicate reactions were performed according to the manufacturer’s instructions. Synthesized cDNA was diluted 1:5 in RNase-free water and 3.0 µl was added to the final reaction (15 µl). The cDNAs were multiplied using Applied Biosystems 7300 Real-time Sequence Detection System: 2 min at 50 °C, 10 min at 95 °C, and 40 cycles repeating denaturation for 15 s at 95 °C and annealing for 1 min at 60 °C. Cycle threshold (Ct) values were determined using 7300 System SDS Software (Applied Biosystems). The 2^ΔΔCt^ method was used to quantify the relative gene expression [30]. Replicate reactions were considered reliable if the standard deviation of the triplicates was less than 0.5. Glyceraldehyde 3-phosphate dehydrogenase (*GAPDH;* Hs99999905_m1) was used as an internal control to normalize the added RNA amount. Downregulation is represented using fold regulation, which is calculated using the following formula: (−1)/fold change.

### Immunostaining

**Figure 3:**
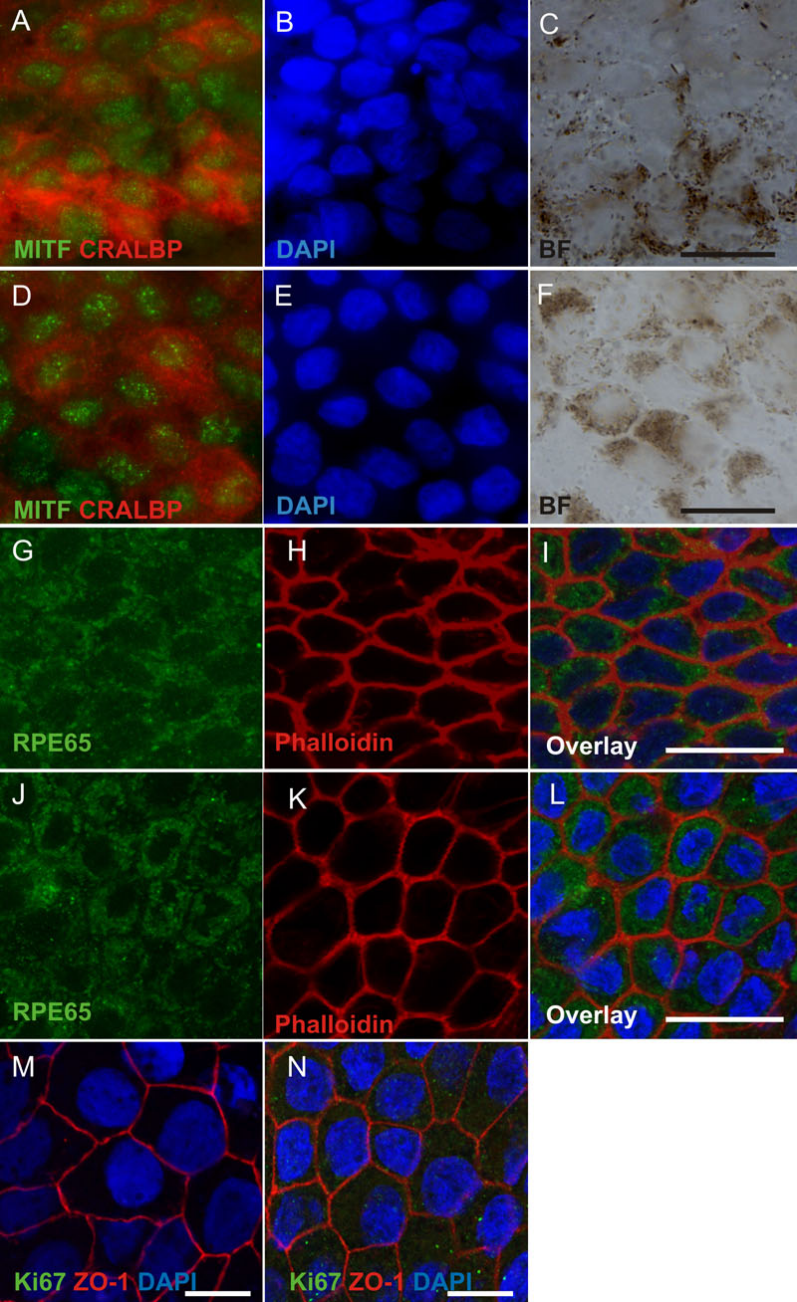
Immunofluorescence staining of human embryonic stem cell (hESC; Regea 08/023)- and human induced pluripotent stem cell (hiPSC; FiPS 5–7)-derived retinal pigment epithelium cells revealing maturation stage after 83 days of differentiation. Cellular retinaldehyde-binding protein (CRALBP) and microphthalmia-associated transcription factor (MITF) localization in **A-C**: manually selected hESC-RPE cells and **D-F**: hiPSC-retinal pigment epithelium (RPE) cells. **G**, **I**: RPE65 expression in hESC-RPE and **J**, **L**: hiPSC-RPE. **H**, **K**: For cell morphology, F-actins were stained using phalloidin. Tight junction protein anti-zonula occludens (ZO)-1 and proliferation marker Ki67 localization in **M**: hESC-RPE cells and **N**: hiPSC-RPE cells. Nuclei were stained with 4',6-diamidino-2-phenylindole (DAPI). Images **A**-**F** were taken with an Olympus BX60 microscope (Olympus, Tokyo, Japan) using a 60× oil immersion objective, scale bar 20 μm. Images **G**-**N** were taken with an LSM 700 confocal microscope (Carl Zeiss) using a 63× oil immersion objective, scale bar 20 μm.

After 51 days of differentiation, putative hESC-RPE and hiPSC-RPE were seeded on permeable Collagen IV–coated 0.3 cm^2^ BD Biocoat culture plate inserts (BD Biosciences) by enzymatic dissociation with 1x Trypsin-EDTA (Lonza Group Ltd, Basel, Switzerland). Cells were maturated on inserts for 32 days. For immunostaining, cells were washed three times with PBS and fixed for 10 min with 4% paraformaldehyde (pH 7.4; Sigma-Aldrich) at room temperature (RT) following repeated washings with PBS. The cells were made permeable by incubating in 0.1% Triton X-100 in PBS (Sigma-Aldrich) at RT for 10 min. Thereafter, the unspecific binding sites were blocked with 3% BSA (BSA; Sigma–Aldrich) at RT for 1 h. Samples were incubated for 1 h at RT with primary antibodies; rabbit anti-microphthalmia-associated transcription factor (MITF) 1:350, rabbit anti-bestrophin 1:500, mouse anti-cellular retinaldehyde-binding protein (CRALBP) 1:1,000, mouse anti-Na^+^/K^+^ATPase 1:50 (all from Abcam, Cambridge, UK), mouse anti-retinal pigment epithelium-specific 65 kDa protein (RPE65) 1:250, rabbit anti-Ki67 1:300 (both from Millipore, Bedford, MA), and mouse anti-zonula occludens 1 (ZO-1) 1:250 (Invitrogen). The cells were washed three times with PBS and labeled with 1:1,500 diluted secondary antibodies: donkey antimouse IgG and goat antirabbit IgG, both Alexa Fluor 488, goat antimouse IgG and goat anti-rabbit IgG, both Alexa Fluor 568 (all from Molecular Probes, Life Technologies, Paisley, UK). Secondary antibodies were diluted in 0.5% BSA-PBS and incubated 1.5 h at RT following repeated PBS washings. In addition, fluorescein isothiocyanate (FITC) phalloidin 1:50 (Invitrogen) was used to label filamentous actin. For clarity, FITC phalloidin is represented as red in Figure 3H,I,K,L and A568 is represented as green in Figure 3G,I,J,L. The nuclei were stained with 4´, 6´ diamidino-2-phenylidole (DAPI) included in the mounting media (Vector Laboratories Inc., Burlingame, CA). Images were taken either with an LSM 700 confocal microscope (Carl Zeiss, Jena, Germany) using a 63× oil immersion objective or Olympus BX60 microscope (Olympus, Tokyo, Japan) using a 60× oil immersion objective.

### In vitro phagocytosis assay

Porcine POS were isolated to study whether the cells have the ability to phagocytose POS [31]. Twenty-three porcine eyes received from a local abattoir were halved with scissors and the retinas were removed using tweezers in a dark room under red light. Retinas were homogenized with homogenizer in 0.73 M sucrose phosphate buffer. Homogenized retinas were filtered and cell types separated in sucrose gradient (0.75 M, 1.0 M, 1.25 M, 1.5 M, 1.75 M) using an ultracentrifuge (Optima ultracentrifuge, Beckman Coulter, Inc., Brea, CA) 60,000× g, 1 h. The pink POS layer was collected in phosphate buffer and centrifuged. For phagocytosis assay, sucrose-phosphate buffer was removed and POS were labeled with FITC (0.04 µg/µl; Sigma-Aldrich) in 0.1M NaHCO_3_ (pH 9) for 1 h at RT, following washing three times with PBS and resuspension in culture medium. Floating cell aggregates, which included pigmented and nonpigmented cells, were incubated with POS for 16 h in a cell culture incubator in culture medium. Subsequently, the cells were washed twice with PBS. Cells were fixed with 4% paraformaldehyde for 30 min at +37 °C following repeated PBS washings. Thereafter, 0.20% Trypan Blue was used to quench external fluorescence following PBS washing. Cells were made permeable using 0.1% Triton X-100 for 10 min at RT followed by repeated PBS washings. Filamentous actin was stained with 1:10 diluted phalloidin 0.02 µg/µl (Sigma-Aldrich) by incubating for 10 min at RT following several PBS washings. The nuclei were stained with DAPI that was in the mounting media (Vector Laboratories Inc.). The images of the RPE cells with intracellular POS fragments were taken using a confocal microscope (LSM 700, Carl Zeiss, 63× oil immersion objective).

### Enzyme-linked immunosorbent assay

The functionality and maturity of putative RPE cells were evaluated by their PEDF secretion. RPEbasic and RPEregES media were conditioned with 10 strongly pigmented floating aggregates each. Secretion of nonpigmented aggregates was used as controls. PEDF secretion was evaluated in four time points; Regea 08/023 was differentiated in RPEbasic conditions for 49, 70, 97, and 230 days, and FiPS 5–7 for 49, 80, 97, and 250 days, respectively. From the RPEregES condition, analyses were conducted with both previously mentioned cell lines on days 49 and 98. Culture media were collected from each well every second or third day. PEDF concentration was determined using Chemikine PEDF Sandwich ELISA Kit according to the instructions of the manufacturer (Millipore). In brief, medium samples were treated with 8 M urea to gain total PEDF secreted in media and incubated on ice for an hour. Urea-treated samples were diluted 1:100 in assay diluent. One hundred µl of diluted samples were added per well of 96 well plate likewise PEDF standards. Incubated for hour at +37 °C following repeated washing with washing buffer. 1:500 diluted Biotinylated Mouse Anti-Human PEDF monoclonal antibody was added into the wells. The plate was incubated for an hour at +37 °C. After repeated washings, 100 µl 1:1000 diluted streptavidin peroxidase conjugate was added to the wells, following hour incubation at +37 °C. TMB/E reagent was added to the wells. Incubated at RT for 5–10 min before addition of Stop Solution, and absorbance was measured immediately using Wallac Victor2™ 1420 Multilabel counter (Perkin Elmer-Wallace, Norton, OH).

### Transepithelial electric resistance

Development of epithelia barrier properties and tight junction formation between the cells reflecting polarity was determined as duplicates from hESC-RPE and hiPSC-RPE cell monolayers cultured on permeable Collagen IV–coated 0.3 cm^2^ BD Biocoat culture plate inserts (BD Biosciences). Human ESC line Regea 08/023 and hiPSC line FiPS 5–7 were differentiated for 178 days and 197 days, respectively, before seeding on the inserts. TEER measurements were taken every time after replacing culture media during 90 day culture period of the monolayer with a Millicell-electrical resistance system volt-ohm meter (Millipore). TEER (Ωcm^2^) of the epithelia was obtained by subtracting the impact of the medium and similarly treated insert without cells from the result and multiplying this by the area of the filter membrane.

### Ethical issues

Regea-Institute for Regenerative Medicine has the approval of the National Authority for Medicolegal Affairs Finland (TEO) to study human embryos (Dnro1426/32/300/05). Regea has the support of the Ethical Committee of the Pirkanmaa Hospital District to derive, culture, and differentiate hESC lines from surplus human embryos (R05116).

## Results

### Comparison of the differentiation rate between cell lines

In this study, we successfully differentiated RPE-like cells from all five human pluripotent stem cell lines that were studied. First, the differentiation potentials of the hESC lines and the hiPSC line were compared by analyzing the appearance of the first pigmented cells emerging from each cell line. On average, pigmentation was observed as follows: Regea 08/013 on D21, both Regea 08/017 and Regea 08/023 on D12, Regea 08/056 on D10, and FiPS 5–7 on D11 (Table 3). The percentage of pigmented cell clusters from each cell line was calculated between day 21 and 28 after the removal of bFGF (RPEbasic). Over 20% of the cell aggregates of the cell lines Regea 08/023, Regea 08/056, and FiPS 5–7 contained pigment cells, whereas less than 10% of the aggregates of the cell lines Regea 08/013 and Regea 08/017 contained pigment cells (Table 3).

**Table 3:** Pigmentation rate of studied cell lines.

**Cell line**	**Appearance of pigmentation± standard deviation (n)**	**Number of pigmented cell aggregates**	**Total number of cell aggregates**	**% Pigmented aggregates**
08/013	d21±3.2 (5)	90	949	9.5
08/017	d12±7.4 (4)	50	775	6.5
08/023	d12±5 (6)	136	582	23.4
08/056	d10±2.1 (2)	55	249	22.1
FiPS 5–7	d11±5.3 (3)	83	333	24.9

n: number of independent experiments

### Eye-specific gene expression during differentiation

**Figure 4:**
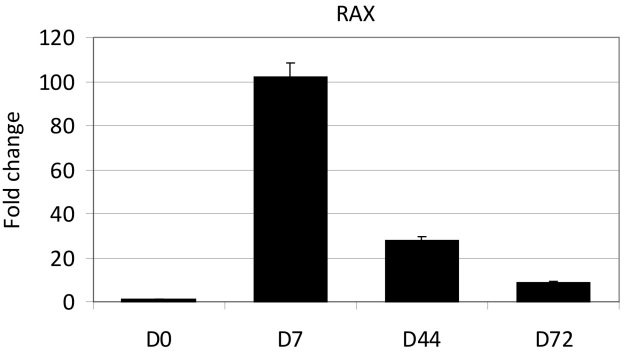
Retina and anterior neural fold homeobox (RAX) expression by FiPS 5–7 during differentiation. Undifferentiated cells (D0) have been used as a reference sample.

Gene expression of the differentiating cells was analyzed using RT–PCR (Table 4). All of the hESC lines expressed the early eye lineage markers paired box gene 6 (*PAX6)* and *RAX*, and early RPE marker *MITF* seven days after removal of bFGF (RPEbasic). In contrast, the expression of *RAX* by the FiPS 5–7 cell line could not be consistently detected until D56. Thus, we further studied *RAX* expression with qRT–PCR. qRT–PCR analyses showed that *RAX* was expressed on D7 and the expression decreased during differentiation (Figure 4). The RPE cell specific markers *RPE65* and Bestrophin were detected from all the cell lines on D7–D28 except for cell line Regea 08/013, from which the expression of *RPE65* was detected for the first time on D44. Other RPE cell markers, orthodenticle homeobox 2 variant 1 (*OTX2v1)*, premelanosome protein (*PMEL)*, and *PEDF* were expressed relatively early during the differentiation process, with tyrosinase showing the latest appearance of the studied genes. Representative RT–PCR results from hESC line Regea 08/023 and hiPSC line FiPS 5–7 are presented in Figure 1B.

**Table 4:** Gene expression profiles during the differentiation.

**Cell line**	**Day**	***GAPDH***	***PAX6***	***RAX***	***MITF***	***RPE65***	**bestrophin**	***OTX2v1***	***PMEL***	***PEDF***	**tyrosinase**
08/013	d7	+	+	(+)	+	-	-	+	+	+	-
08/013	d28	+	+	+	+	-	+	+	+	+	-
08/013	d44	+	+	+	+	+	+	(+)	+	+	+
08/013	d52	+	+	+	+	+	+	(+)	+	+	+
08/013	d72	+	+	+	+	+	+	(+)	+	+	+
08/017	d7	+	+	+	+	-	-	+	(+)	+	-
08/017	d28	+	+	(+)	+	+	+	+	+	+	-
08/017	d44	+	+	+	+	+	+	+	+	+	-
08/017	d52	+	+	-	+	+	+	+	+	+	+
08/017	d72	+	+	+	+	+	+	+	+	+	+
08/023	d7	+	+	+	+	-	(+)	+	+	+	-
08/023	d28	+	+	+	+	+	(+)	+	+	+	-
08/023	d44	+	+	+	+	+	+	+	+	+	(+)
08/023	d52	+	+	+	+	+	+	+	+	+	(+)
08/023	d72	+	+	+	+	+	+	+	+	+	(+)
08/056	d7	+	+	(+)	+	+	+	+	+	+	-
08/056	d27	+	+	+	+	+	+	+	+	+	+
08/056	d44	+	+	+	+	+	+	+	+	+	+
08/056	d54	+	+	+	+	+	+	+	+	+	+
08/056	d72	+	+	+	+	+	+	+	+	+	+
FiPS 5–7	d7	+	+	-	+	-	+	+	+	+	-
FiPS 5–7	d28	+	+	-	+	+	+	+	+	+	-
FiPS 5–7	d44	+	+	-	+	+	+	(+)	+	+	-
FiPS 5–7	d56	+	+	(+)	+	+	+	+	+	+	+
FiPS 5–7	d72	+	+	+	+	+	+	+	(+)	+	(+)

In the table + means positive expression, (+)means faint expression, - means negative expression. Abbreviations: Glyceraldehyde 3-phosphate dehydrogenase (*GAPDH*), paired box gene 6 (*PAX6*), retina and anterior neural fold homeobox (*RAX*), microphthalmia-associated transcription factor (*MITF*), anti-retinal pigment epithelium-specific 65 kDa protein (*RPE65*), orthodenticle homeobox 2 variant 1 (*OTX2v1*), premelanosome protein (*PMEL*), pigment epithelium-derived factor (*PEDF*).

### Pigmented cells expressed retinal pigment epithelium–specific genes and proteins

**Figure 5:**
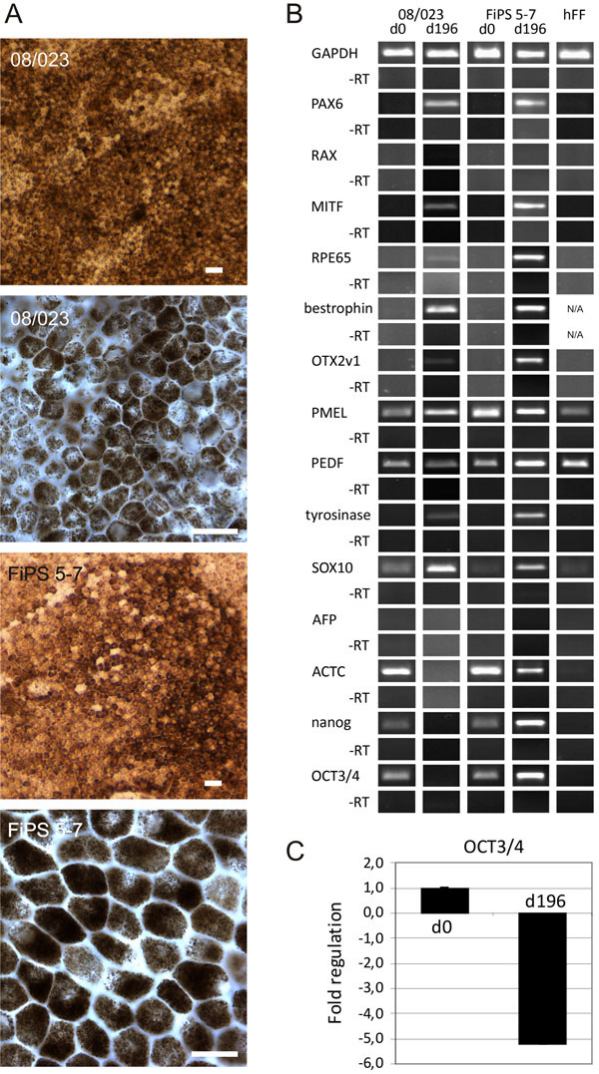
Morphology and gene expression analysis of manually selected and long-term cultured human embryonic stem cell (hESC)-retinal pigment epithelium (RPE; Regea 08/023) and hiPSC-RPE (FiPS 5–7) cells. **A**: Bright-field micrograph of hESC-retinal pigment epithelium (RPE) and human induced pluripotent stem cell (hiPSC)-RPE cells cultured for 136 days on Collagen IV. The cells have acquired a cobblestone morphology and a high degree of pigmentation, which is typical of RPE cells. Low magnification images were captured with a Nikon Eclipse TE2000-S phase contrast microscope (Nikon Instruments Europe B.V. Amstelveen, The Netherlands) and higher magnification images with an Olympus BX60 microscope (Olympus, Tokyo, Japan) using a 60× oil immersion objective. Scale bar 20 μm. **B**: Reverse transcription (RT)–PCR analysis showing the expression of optic vesicle, optic cup, RPE, neural crest melanocyte, pluripotent stem cell, mesoderm and endoderm marker genes by undifferentiated cells (D0), human foreskin fibroblast (hFF) feeder cells, and putative RPE cells (D196) from Regea 08/023 and FiPS 5–7 cells. N/A=not analyzed. **C**: Relative OCT3/4 expression between undifferentiated FiPS 5–7 and putative hiPSC-RPE after 196 days of differentiation.

According to the pigmentation rate (Table 3), we chose the hESC line Regea 08/023 and hiPSC line FiPS 5–7 for more detailed evaluation. The isolated pigmented cells were matured on Collagen IV–coated well plates. Morphologically, cultured cells seemed to undergo an epithelial-mesenchymal transition (EMT) process; after attachment, cells had fibroblast-like morphology following transition to round cells and finally to morphologically typical cobblestone-like cells, putative RPE cells with pigment granules (Figure 5A). The isolated and long-term cultured (d196) hESC- and hiPSC-derived RPE-like cells expressed the typical RPE cell genes (*MITF*, *RPE65*, bestrophin, *OTX2v1*, *PMEL*, *PEDF*, and tyrosinase; Figure 5B). Of the studied genes, undifferentiated cells showed the expression of *PMEL*, *PEDF*, *SOX10*, alphacardiac actin, *nanog*, and *OCT3/4*. hFF used in undifferentiated cell cultures showed the expression of *PMEL*, *PEDF*, and *SOX10* (Figure 5B). In addition, the cells expressed eye precursor gene *PAX6*; however, *RAX* showed only very faint expression. The endodermal marker alphafetoprotein was not expressed in long-term cultured cell population, but mesodermal marker alphacardiac actin was expressed by hiPSC-RPE cells and a marker of neural crest-derived melanocytes (*SOX10*) was expressed by both cell lines. In addition, the expression of the pluripotent markers nanog and *OCT3/4* was detected from hiPSC-RPE but was absent in hESC-RPE cells (Figure 5B). However, qRT–PCR comparison between undifferentiated FiPS 5–7 cells and putative hiPSC-RPE cells showed that the expression level was very low on D196 of differentiation (Figure 5C).

To confirm that the manually isolated cells expressed proteins typical of functional RPE, the expression and localization of MITF, CRALBP, RPE65, and ZO-1 were analyzed with immunostaining (Figure 3). MITF localized in the nuclei and CRALBP both to the cytoplasm and cell membrane (Figure 3A,D), as expected. RPE65 was observed in cytoplasm. Phalloidin staining showed F-actin distribution adjacent to the cell membrane, resembling native RPE cells (Figure 3H,K). ZO-1 localized to the tight junctions on the cell membrane and K_i_-67 staining was negative, suggesting that cells were mature and did not proliferate at this stage (Figure 3M,N).

### Functional characterization of differentiated cells

**Figure 6:**
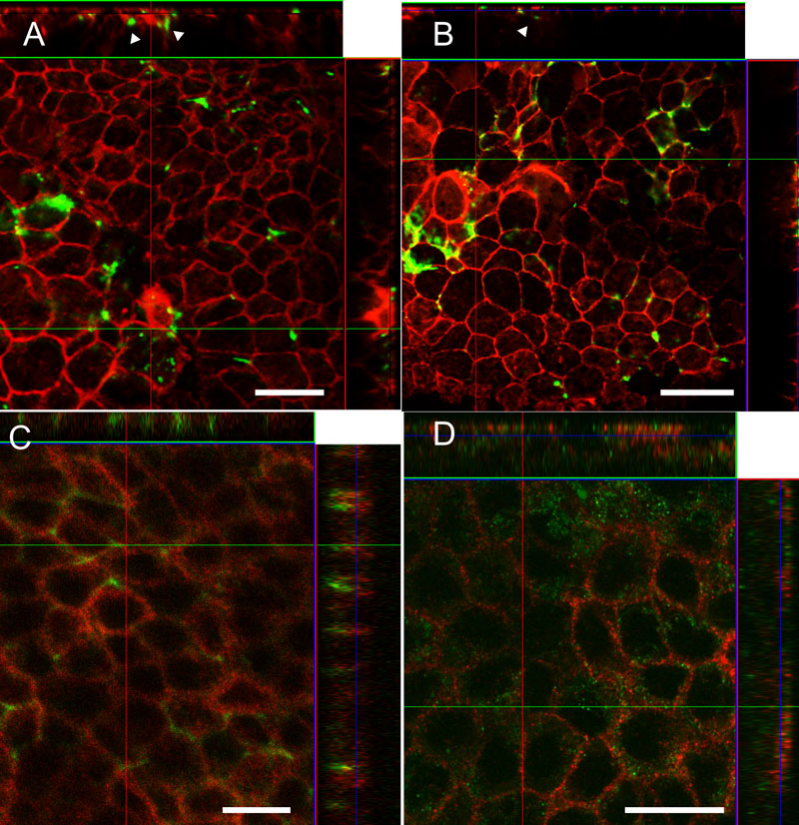
Phagocytosis of photoreceptor outer segments (POS) and cell membrane polarization of human embryonic stem cell (hESC; Regea 08/023) and human induced pluripotent stem cell (hiPSC; FiPS 5–7)-derived retinal pigment epithelium (RPE) cells. **A**: Putative hESC-RPE and **B**: hiPSC-RPE internalize POS (green, arrowheads) for cell morphology; F-actins were stained using phalloidin (red). Vertical confocal sections showing apical localization of Na^+^/K^+^ATPase (green) and basolateral localization of Bestrophin (red) in **C**: hESC-RPE and **D**: hiPSC-RPE. Images were taken with an LSM 700 confocal microscope (Carl Zeiss) using a 63× oil immersion objective, scale bar 20 μm.

The functionality of putative hESC-RPE (Regea 08/023) and hiPSC-RPE (FiPS 5–7) was shown by POS phagocytosis, PEDF secretion, polarization of cells, and the integrity of the epithelial structure by TEER measurements. The results show that pigmented cells from both lines were able to phagocytose POS (Figure 6A,B), as opposed to nonpigmented cells, which were used as controls (data not shown).

Pigmented cell aggregates differentiated with RPEbasic method also secreted PEDF to the culture medium, whereas nonpigmented cells did not. On average, PEDF secretion after 49 days of differentiation was 1.7 ng/ml from putative hESC-RPE (Regea 08/023) and 13.8 ng/ml from putative hiPSC-RPE (FiPS 5–7). On D70, the secretion was increased to 77.5 ng/ml from putative hESC-RPE (Regea 08/023) cells and 44.8 ng/ml from putative hiPSC-RPE (FiPS 5–7) cells on D80, respectively. After 97 days of differentiation, PEDF secretion was 548.0 ng/ml (Regea 08/023) and 251.8 ng/ml (FiPS 5–7). After 230 days of differentiation, secretion was 434 ng/ml from hESC-RPE cells. From hiPSC-RPE-like cells, secretion was 15.5 ng/ml on D250. It must be noted that the exact cell amount was not the same between media collections. hFF cells, which were used as feeder cells for pluripotent cells, also showed PEDF expression at the gene level, but did not secrete a detectable amount of PEDF to the culture medium (data not shown).

The polarization of the cells was analyzed by localization of Na^+^/K^+^ATPase and bestrophin proteins. Na^+^/K^+^ATPase was localized on the apical cell membrane and Bestrophin on the basolateral side, demonstrating the polarization of analyzed cells (Figure 6C,D).

The development of the integrity of the epithelia was assessed weekly during a 90 day period after seeding the cells on porous culture inserts. Thirty days after plating the pigmented cells on the inserts, TEER values ranged between 6 and 32 Ωcm^2^ and were 6–10 Ωcm^2^ in hESC-RPE (Regea 08/023) and hiPSC-RPE (FiPSC 5–7), respectively. After 60 days on inserts, TEER values ranged between 145 and 188 Ωcm^2^ for putative hESC-RPE and were 23–38 Ωcm^2^ for putative hiPSC-RPE. After 90 days on the inserts, TEER values for hESC-RPE reached 311 Ωcm^2^ and for hiPSC-RPE, 74 Ωcm^2^.

### Xeno-free and defined conditions induce efficient retinal pigment epithelium differentiation

Finally, we wanted to evaluate RPE differentiation in defined and xeno-free conditions (RPEregES) and to compare this with our findings in basic conditions (RPEbasic). The results from a pilot study with the Regea 08/013 cell line, which was derived and maintained in RegES [27], indicated that hESCs derived and maintained in RegES are able to spontaneously differentiate into pigmented cells resembling RPE morphology when bFGF is excluded (data not shown). To compare the RPEregES method with RPEbasic, we implemented further studies with Regea 08/023 and FiPS 5–7. The first pigmented cells were observed in hESC line Regea 08/023 on D7 and in hiPSC line FiPS 5–7 on D6 in RPEregES conditions, compared to D12 and D11, respectively, in RPEbasic. The ratio of the pigmented cell clusters (n) was evaluated after 23–28 days of differentiation. The percentage of the pigmented clusters for FiPS 5–7 cells was 37% (n=129) in RPEregES conditions and 25% (n=457) in RPEbasic conditions, and for Regea 08/023 cells 43% (n=129) in RPEregES and 25% (n=645) in RPEbasic conditions.

**Figure 7:**
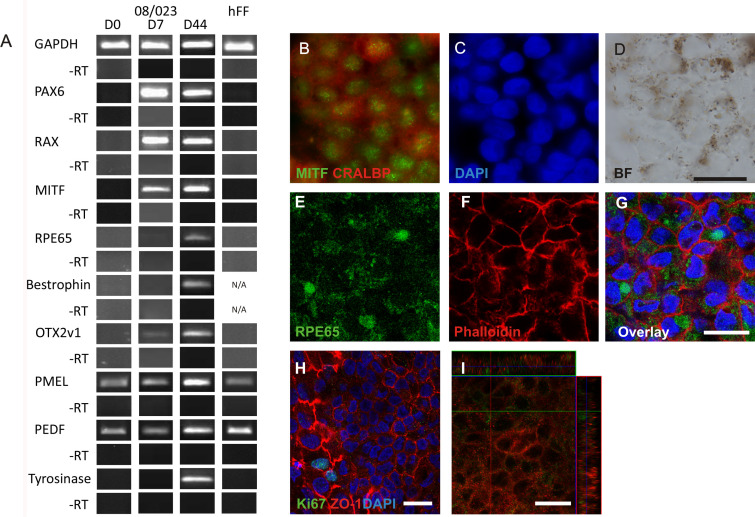
Differentiation of human pluripotent stem cells toward retinal pigment epithelium (RPE) cells under defined culture conditions, RPEregES. All represented images are from human embryonic stem cell (hESC)-RPE Regea 08/023. **A:** Reverse transcription (RT)–PCR analysis of typical genes for retinal/ RPE development expressed by undifferentiated hESC (Regea 08/023), human foreskin fibroblast (hFF) feeder cells, and putative hESC-RPE on D7 and D44. Expression of **B**: Microphthalmia-associated transcription factor (MITF), **B**: Cellular retinaldehyde-binding protein (CRALBP), and **E**,**G**: RPE65 on D83. **F**: For cell morphology, F-actins were stained using phalloidin. **H**: Proliferative activity was studied by Ki67 staining together with tight junction protein anti-zonula occludens (ZO)-1 in hESC-RPE. **I**: Vertical confocal sections showing apical localization of Na^+^/K^+^ATPase (green) and basolateral localization of Bestrophin (red). Nuclei stained with 4',6-diamidino-2-phenylindole (DAPI). Images **B**-**D** were taken with an Olympus BX60 microscope (Olympus, Tokyo, Japan) using a 60× oil immersion objective, scale bar 20 μm. Images **E**-**I** were taken with an LSM 700 confocal microscope (Carl Zeiss) using a 63× oil immersion objective, scale bar 20 μm.

RT–PCR and immunostainings were performed for the cells (Regea 08/023 and FiPS 5–7) differentiated in RPEregES medium. On D44, cells from both cell lines expressed all analyzed eye/RPE-specific markers (*PAX6, RAX, MITF, RPE65, *bestrophin*, OTX2v1, PMEL, PEDF, *tyrosinase; Regea 08/023, Figure 7A; FiPS 5–7, data not shown). The pigmented cells in RPEregES conditions were manually selected on D51. Their morphology and protein expression were analyzed on D83. The pigmented cells differentiated from both hESC and hiPSC lines had cobblestone-like morphology, which is typical for RPE cells. In addition, the cells were positive for RPE65, ZO-1, MITF, CRALBP, Bestrophin, and Na^+^/K^+^ATPase, which are important for the functionality of the RPE cell. There were some Ki67 positive cells, which were clearly more immature according to the cell morphology (Regea 08/023, Figure 7B-I; FiPS 5–7, data not shown). In addition to gene and protein expression, putative RPE cells differentiated with RPEregES secreted PEDF. On average, PEDF secretion after 49 days of differentiation was 16.2 ng/ml and 97 days was 182.4 ng/ml from hESC-RPE (Regea 08/023) cells. Corresponding values from hiPSC-RPE (FiPS 5–7) differentiated with the RPEregES method were 10.6 ng/ml and 463.15 ng/ml.

## Discussion

In this study, we successfully differentiated RPE-like cells from several human pluripotent stem cell lines without the use of animal cells or serum during the differentiation. In addition, we reported RPE differentiation in xeno-free and defined culture conditions. Putative RPE cells arise spontaneously from undifferentiated cells by the removal of bFGF from the culture conditions. The hESC lines used in this study have a similar background; all the cell lines were cultured on hFF feeder cells and in KO-SR containing culture medium without serum. The hiPSC line, FiPS 5–7, was cultured on top of hFF feeder cells from passage 26 onwards. Other published RPE differentiation methods have harnessed mainly hESC and hiPSC lines derived and cultured on MEFs or with FBS (Table 1). Only very recently, Idelson and coworkers published a protocol differentiating hESCs toward RPE cells from hESCs grown on hFF feeder cells and with KO-SR containing culture medium [16].

Our results demonstrate that the appearance of the first pigmented cells was relatively fast after the removal of bFGF, both in hESC and hiPSC lines, varying from 10 to 21 days. Most of the published data describe the appearance of the first pigmented cells usually around 2 to 8 weeks [1,32,33]. Thus, according to their pigmentation, hESCs and hiPSCs cultured on hFF seem to have equally good differentiation rate to those of other published cell lines cultured on MEFs. We analyzed the percentage of the pigment containing cell clusters to indicate the differentiation rate of each cell line on D21–D28 after initiation of the differentiation. Although calculations only give a rough estimate of the pigmentation rate, the results demonstrated differences between analyzed cell lines. Idelson and coworkers published comparable results with an equal amount of pigmented cell clusters (<15%) after four weeks of differentiation without NIC supplementation [16]. For comparison, Klimanskaya and coworkers reported the presence of pigmented islands in less than 1% of hESC-derived cell aggregates after 4–8 weeks of differentiation [1].

During the time course of differentiation, we already detected the expression of early eye lineage markers *PAX6* and *RAX*, as well as early RPE marker *MITF* at D7 after removal of bFGF from all analyzed hESC lines. Although the expression of *PAX6* was also detected during a later stage of the differentiation, our data demonstrated that the mature RPE cell specific markers, *RPE65* and bestrophin, were detected from all but one of the hESC lines on D28 at the latest. In addition, other RPE cell markers—*OTX2v1, PMEL, PEDF*, and tyrosinase—were also expressed early in the differentiation process of hESC lines, with tyrosinase having the latest appearance of the studied genes. Surprisingly, the expression of *RAX* was not detected from hiPSC-RPE (FiPS 5–7) until D56. On the other hand, RPE cell specific markers *RPE65, *bestrophin*, OTX2v1 PMEL, PEDF*, and tyrosinase were expressed in hiPSC-RPE derived cells similarly as to hESC-RPE cells.

According to the pigmentation rate, we chose the most promising hESC line, Regea 08/023, and hiPSC line, FiPS 5–7, for more detailed evaluation. Manually selected and long-term cultured cells formed a monolayer of pigmented cobblestone-like cells that had a very similar morphology to previously published hESC-RPE and hiPSC-RPE cells [1,32,34]. After dissociation into single cells, the differentiated RPE-like cells started to proliferate and were observed to undergo morphological changes that are typical in EMT. EMT is related to normal development and tissue repair, but also pathological processes such as cancer and proliferative vitreoretinopathy. The EMT process has been recently described in isolated RPE cells [35]; thus it is a natural feature when RPE cells lose cell-cell contacts. Our results indicate that putative hESC-RPE and hiPSC-RPE cells also seem to undergo a similar process.

The gene expression analyses of hESC-RPE and hiPSC-RPE cells revealed that the cells expressed eye precursor genes *RAX* and *PAX6*, and RPE cell markers. The expression of *RAX* was not constantly detected from hiPSC-RPE (FiPS 5–7) until D56 with RT–PCR. However, more quantitative qRT–PCR analysis showed that *RAX* expression was similar to that of hESC-RPE cells. Both cell lines also expressed the marker of neural crest-derived melanocytes (*SOX10*), indicating the presence of other ectoderm derivatives. *SOX10* expression by selected hESC and hiPSC derived RPE-like cells is most probably the expression of other types of neural cells, which easily contaminate pluripotent human cell cultures [36]. Selected and long-term cultured hiPSC-RPE cells expressed mesodermal marker and pluripotent marker *OCT3/4* and nanog, indicating that the cell population contained undifferentiated cells. We showed that neither hESC-RPE nor hiPSC-RPE were homogenous, even after 196 days in culture, according to gene expression, although morphologically the cells seemed to be of uniform quality. Thus, the manual selection, which we used in this study, is not sufficient to gain a pure population of putative RPE cells. Consequently, it is essential to develop more specific differentiation methods and more efficient purification and selection methods for these cells.

Microscopy of cells differentiated for 83 days showed that the cells were highly organized and pigmented. The immunocytochemical localization of proteins essential for mature and functional RPE cells was identical to previously described results [16,37]. Of note, the cells expressed RPE65, which is essential for the regeneration of the visual pigment required for both rod- and cone-mediated vision [38]. The expression of the RPE65 protein is typically lost in cultured RPE cells [39-41], and has been reported in only a few papers describing the differentiation of RPE cells from human ESCs [16,37]. The expression of RPE65 protein in a culture environment by human iPSC-RPE has been previously reported only by western blotting and was detected as late as after eight months of differentiation [32]. In the present study, both hESC-RPE and hiPSC-RPE cells showed RPE65 immunostaining at D83, suggesting that the differentiated cells are functional and closely resemble the native RPE cells.

One of the most important functions of the RPE cells is the phagocytosis of POS. In vivo, photoreceptor cells undergo a daily renewal process and RPE cells take care of the waste disposal by phagocytosing nonfunctional POS. Several groups have published the phagocytotic activities of hESC-RPE and hiPSC-RPE in vitro using latex beads [1] or POS isolated from animals [32,42]. It has been further suggested that the only proof for the specific phagocytosis activity of RPE cells is the capability for POS phagocytosis [42]. The hESC-RPE and hiPSC-RPE cells generated in our study possess the relevant molecular functions required for the phagocytosis of isolated POS, thus demonstrating their functionality in vitro.

In addition, the functionality of hESC-RPE and hiPSC-RPE cells was shown by PEDF secretion, with both differentiation methods described here. PEDF secreted by RPE cells is antiangiogenic and neuroprotective, protecting retinal neurons from light damage, oxidative stress, and glutamate excitotoxicity [43]. According to our knowledge, the secretion of PEDF from putative hESC-RPE or hiPSC-RPE cells has not been previously published anyone other than Klimanskaya and coworkers [1]. Our data demonstrated that the differentiated pigmented cells were able to secrete high levels of PEDF into the culture medium, while nonpigmented cells did not secrete any detectable amounts of PEDF. There is no published data about the amount of PEDF secreted by putative hESC/hiPSC-RPE [1]. Tong and coworkers have reported that the ARPE19 line secretes PEDF at around the 5–35 ng/ml level to the culture medium [44], and Maminishkis and coworkers have stated that isolated human fetal RPE cells secrete PEDF at the 600 ng/ml level [45]. These levels have not been standardized to the cell number included in the experiment; thus, direct comparison of our results is not possible. However, the hESC-RPE and hiPSC-RPE cells in our study secrete reasonable levels (40 ng/ml- 430 ng/ml) of PEDF, demonstrating their functionality in relation to growth factor production in vitro.

Versatile determination of the maturation and polarization capacity of the derived cells in a specific culture protocol is important [32]. TEER is one of the assessments that can be used for this [46]. TEER values have been regularly assessed from human retinal explants [47] and primary and immortalized RPE cell lines reaching 206 Ωcm^2^ and 100 Ωcm^2^, respectively [48,49]. To our knowledge, the TEER of putative hESC-RPE or hiPSC-RPE cells has not yet been evaluated. In our culture, the putative hESC-RPE cells reached TEER values of 310 Ωcm^2^ after 268 days of differentiation and the putative hiPSC-RPE cells reached TEER values of 74 Ωcm^2^ after 287 days of differentiation. When the TEER values of hESC/hiPSC-RPE are compared to values derived from other RPE cell cultures, it appears that putative hESC-RPE cells are at least as good or even superior to immortalized RPE cell lines in forming a highly polarized monolayer. Further indications of a high degree of cellular polarization of differentiated cells are the separation of Na^+^/K^+^ATPase to the apical and bestrophin to the basal side of the cellular monolayer, and junctional localization of apical tight junction protein ZO-1.

We further demonstrated that xeno-free and defined differentiation conditions can be used for the induction and maturation of pluripotent stem cell derived RPE cells. With this method, we were able to differentiate RPE-like cells from hESCs and hiPSCs. The differentiated cells showed similar gene and protein expression profiles, as well as cellular polarization, to the RPE cells differentiated using RPEbasic conditions containing xenogeneic substances. Our results demonstrated that the appearance of the pigmented cells was slightly faster in RPEregES conditions as compared to the cells in RPEbasic conditions. In addition, the percentage of the pigmented clusters after 23 days of differentiation of both the hESC and hiPSC lines were higher in RPEregES conditions, with the hiPSC line having over 10% difference between the two conditions. These results suggested that our defined and xeno-free culture medium described previously [27] may even enhance the differentiation of RPE cells. This may be partly explained by the fact that the medium contains Activin A (5 ng/ml), a known inducer of RPE cell fate [26]. Our defined and xeno-free differentiation method is one step forward when optimizing proper culture conditions for clinically eligible RPE cells for transplantation. It remains to be studied whether supplements such as NIC [16] and Wnt and Nodal antagonists [21,22] increase RPE differentiation from hESCs and hiPSCs in our culture systems.

In conclusion, we have demonstrated a progressive differentiation protocol for the production of functional RPE-like cells from hESCs and hiPSCs. Our results demonstrate that putative hESC-RPE and hiPSC-RPE express genes and proteins characteristic for RPE cells, can phagocytose POS, are able to secrete PEDF and form highly polarized and tight epithelial structures in vitro. Our results show that highly mature RPE-like cells can be differentiated in xeno-free and defined culture systems that are easier to translate under the Good Manufacturing Practice production systems needed for clinical use. Furthermore, defined conditions will greatly elucidate the further development of more efficient differentiation protocols and the use of cells in drug screening and toxicology studies.
